# History of lower-limb complications and risk of cancer death in people with type 2 diabetes

**DOI:** 10.1186/s12933-020-01198-y

**Published:** 2021-01-04

**Authors:** Kamel Mohammedi, Stephen Harrap, Giuseppe Mancia, Michel Marre, Neil Poulter, John Chalmers, Mark Woodward

**Affiliations:** 1 Department of Endocrinology, Diabetes and Nutrition, Bordeaux University Hospital, Bordeaux, France; 2 Faculty of Medicine, The University of Bordeaux, Bordeaux, France; 3 INSERM Unit 1034, Bordeaux, France; 4 The University of Melbourne and Royal Melbourne Hospital, Melbourne, VIC Australia; 5 The University of Milan-Bicocca and Istituto Auxologico Italiano, Milan, Italy; 6 Université de Paris, UFR de Médecine, Paris, France; 7 Centre de Recherche Des Cordeliers, INSERM, Sorbonne Université, Université de Paris, Paris, France; 8 CMC Ambroise Paré, Neuilly-sur-Seine, France; 9 The International Centre for Circulatory Health, National Heart and Lung Institute, Imperial College, London, UK; 10 The George Institute for Global Health, Sydney, NSW Australia; 11 The University of New South Wales, Sydney, NSW Australia; 12 Faculty of Medicine, Imperial College London, London, UK; 13 Department of Epidemiology, Johns Hopkins University, Baltimore, MD USA

**Keywords:** Cancers, Cancer death, Lower-limb complication, Peripheral arterial disease, Peripheral neuropathy, Type 2 diabetes

## Abstract

**Background:**

Individuals with diabetes and lower-limb complications are at high risk for cardiovascular and all-cause mortality, but uncertainties remain in terms of cancer-related death in this population. We investigated this relationship in a large cohort of people with type 2 diabetes.

**Methods:**

We used data from the Action in Diabetes and Vascular Disease: PreterAx and DiamicroN Modified-Release Controlled Evaluation (ADVANCE) study. The primary outcome was adjudicated cancer death; secondary outcomes were overall and site-specific incident cancers, determined according to the International Classification of Diseases Code (ICD-10). We compared outcomes in individuals with (*versus* without) a baseline history of lower-limb complications (peripheral artery disease (PAD) or sensory peripheral neuropathy) using Cox regression models.

**Results:**

Among 11,140 participants (women 42%, mean age 66 years), lower-limb complications were reported at baseline in 4293 (38%) individuals: 2439 (22%) with PAD and 2973 (27%) with peripheral neuropathy. Cancer death occurred in 316 (2.8%) participants during a median of 5.0 (25th–75th percentile, 4.7–5.1) years of follow-up corresponding to 53,550 person-years and an incidence rate of 5.9 (95% CI 5.3–6.6) per 1000 person-years. The risk of cancer death was higher in individuals with (*versus* without) lower-limb complication [hazard ratio 1.53 (95% CI, 1.21–1.94), *p* = 0.0004], PAD [1.32 (1.02–1.70), *p* = 0.03] or neuropathy (1.41 (1.11–1.79), *p* = 0.004], adjusting for potential confounders and study allocations. PAD, but not neuropathy, was associated with excess risk of incident cancers.

**Conclusions:**

PAD and peripheral neuropathy were independently associated with increased 5-year risk of cancer death in individuals with type 2 diabetes. PAD was also associated with increased risk of incident cancers. Our findings provide new evidence on the non-cardiovascular prognostic burden of lower-limb complications in people with type 2 diabetes.

## Background

Peripheral artery disease (PAD) and peripheral sensory neuropathy are major lower-limb complications frequently observed in patients with diabetes [1–5]. These are the leading causes of non-traumatic lower-extremity amputation, at least 8 times more common in people with diabetes than in persons without diabetes [6, 7]. Lower-limb complications are associated with major disability, worsening quality of life and huge impacts on health care systems and societies [1, 8, 9]. They are also associated with a dramatic reduction in life expectancy, despite major improvements in medical care during recent decades [3, 10–13]. The excess risk of premature death observed in patients with diabetes and lower-limb complications may not be completely attributable to cardiovascular disease, as other non-cardiovascular conditions may also be involved [14].

Cancer is a major public health problem worldwide, and is the second leading cause of death [15]. Growing data suggest an excess risk for cancer death among adults with diabetes, compared with others [16–19]. The risk of cancer increased in people with cardiovascular disease as did the risk of cardiovascular disease in cancer patients [20]. Only few studies have evaluated the risk for cancer death according to vascular complications in people with diabetes, and have reported inconsistent findings [21–23]. As far as we are aware, the relationship between lower-limb complications and cancer death has not been investigated in patients with diabetes. In the present study, we aimed to evaluate the incidence and the relative risk of cancer death according to the baseline history of lower-limb complications among patients with type 2 diabetes in the Action in Diabetes and Vascular Disease: PreterAx and DiamicroN Modified-Release Controlled Evaluation (ADVANCE) study.

## Methods

### Participants

ADVANCE was a large multicentre international randomized trial conducted in patients with type 2 diabetes to test the effects of intensive glucose control using a gliclazide-MR based regimen and blood pressure treatment using a fixed-dose combination of perindopril and indapamide on the incidence of major microvascular and macrovacular events [24]. The design and clinical characteristics of participants in ADVANCE have been published previously [24–26]. Briefly, patients with type 2 diabetes mellitus, and at least one additional risk factor for cardiovascular disease, were randomly assigned in a 2 X 2 factorial design to: (i) gliclazide (modified release)–based intensive glucose-control regimen, targeting an HbA1c of ≤ 6.5%, or to standard glucose control, with targets and regimens based on local guidelines, and (ii) a fixed-dose combination of perindopril (4 mg) and indapamide (1.25 mg) or matching placebo. Participants were enrolled between 2001 and 2003; they were seen 3, 4, and 6 months after randomisation, and subsequently, every 6 months until June 2007 (for blood-pressure–lowering comparison) [25]. The follow-up in the randomized glucose-control regimen was continued for an additional 6 months, until January 2008 with a different follow-up schedule as published previously [25, 26]. The protocol of the ADVANCE trial was approved by the Institutional Ethics Committee of each participating centre and all participants provided written informed consent.

### Definition of lower-limb complications at baseline

A history of lower-limb complication was defined as the presence at baseline of PAD or peripheral neuropathy. PAD was defined as the presence of at least one of following conditions: lack of peripheral pulse (dorsalis pedis or posterior tibial) palpation, requirement of lower-limb revascularisation (surgery, angioplasty or emergency thrombolysis), or lower-extremity amputation of at least one digit, thought to be due to arterial insufficiency. Peripheral sensory neuropathy was defined as the presence at baseline of at least two neurological abnormalities [27]: disturbance of the light touch sensation, abolition of ankle or knee reflex or chronic (at least 6 weeks) foot ulceration. Data were collected by physicians for each participant based on interview and clinical examination, and reported in the case report forms.

### Definition of other conditions at baseline

History of coronary artery disease (CAD) was defined as the presence at baseline of at least myocardial infarction, coronary artery bypass graft, percutaneous transluminal coronary angioplasty, or hospital admission for unstable angina. History of cerebrovascular disease was defined as the presence at baseline of stroke or transient ischaemic attack. Diabetic retinopathy was defined as the presence of at least one of the following conditions: proliferative retinopathy, macular oedema, requirement of retinal laser photocoagulation therapy, or diabetes-related blindness. History of diabetic kidney disease (DKD) was defined as urinary albumin-to-creatinine ratio (ACR) > 30 µg/mg or estimated glomerular filtration rate (eGFR, computed using the Chronic Kidney Disease Epidemiology Collaboration equation) < 60 ml/min/1.73 m^2^.

Region of origin was categorized as 3 groups: Asia (Philippines, China, Malaysia, and India), established market economies (Australia, Canada, France, Germany, Ireland, Italy, Netherlands, New Zealand, and United Kingdom) and Eastern Europe (the Czech Republic, Estonia, Hungary, Lithuania, Poland, Russia, and Slovakia). Cognitive function was estimated by the mini-mental state examination (MMSE) score and considered as normal (MMSE score ≥ 28) or reduced (MMSE score < 28). Education accomplishment was defined as age at completion of the highest level of formal education, and categorized as basic (≥ 16 years) or low (≤ 15 years).

### Primary and secondary endpoints

The primary endpoint for this analysis was cancer death occurring during follow-up. The cause of each death, including cancer-related death, was adjudicated by an independent endpoint committee, blinded to study allocations. The adjudication committee reviewed source documentations for all individuals who died during follow-up. The secondary endpoints were overall and site-specific incident cancers, defined as the first cancer diagnosed after entry to the study according to the International Classification of Diseases Code Tenth Revision (ICD-10). Diagnostic codes used in the study are presented in Additional file 1: Table S1. Incident cancers were collected systematically for all participants during the scheduled study visits from case report forms, and from reports of serious adverse events, without adjudication. Information about the occurrence of all serious adverse events was reported at the time of occurrence between visits. When serious adverse events occurred, the responsible investigator of each centre ensured that the event was reported immediately by completing a Serious Adverse Events Form. The Data and Safety Monitoring Committee regularly reviewed all such events for each centre. The effect of intensive (*versus* conventional) glucose control on cancer death in ADVANCE has been previously reported [28].

### Statistical analyses

Quantitative variables were expressed as mean (SD), or median (25th, 75th percentiles) for those with skewed distributions. Categorical parameters were expressed as numbers and percentages.

Cox proportional hazards regression models were used to estimate hazard ratios (HR) and their 95% confidence intervals (CI) for risk of outcomes according to our three index exposures. We compared the incidence of cancer death according to study allocations: perindopril/indapamide combination (*versus* placebo) or both intensive glucose control and active blood pressure treatment (*versus* standard glucose control and placebo group).

Kaplan–Meier curves were used to plot the cumulative incidence of cancer death during follow-up according to the baseline history of lower-limb complication, PAD or peripheral neuropathy at baseline. Survival curves were compared using the log-rank test. Cox proportional hazards regression models were fitted to estimate the risk of outcomes during follow-up according to the history of lower-limb complication at baseline. Analyses were adjusted for baseline age, sex, region of origin and study allocations (model 1), and for model 1 plus any potential confounders: duration of diabetes, body mass index, waist circumference, systolic and diastolic blood pressure, HbA1c, urinary ACR, eGFR, total cholesterol, HDL cholesterol, triglycerides, MMSE score, education accomplishment, history of ever or current smoking, history of past or current alcohol drinking, history of CAD, cerebrovascular disease, diabetic retinopathy or dementia, and use of metformin, insulin, antihypertensive, statins or antiplatelet therapies (model 2). Continuous variables supposed to have U-shaped relationships with death (age, body mass index, waist circumference, systolic and diastolic blood pressure, HbA1c, and eGFR) were introduced within the model with their squared values.

We tested for interaction between PAD and peripheral neuropathy in their association with cancer death. The proportional hazards assumption was checked using the Schoenfeld residuals method.

We performed a series of sensitivity analyses to evaluate the risks of endpoints according to: 1/ the history of lower-limb complications at baseline using an alternative definition of lower-limb complications: the presence of at least one of the following conditions: PAD (lack of peripheral pulse palpation or requirement of lower-limb revascularisation), peripheral neuropathy (at least two neurological abnormalities: disturbance of the light touch sensation, abolition of ankle or knee reflex), or tissue or limb loss (foot ulceration or lower-extremity amputation); 2/ peripheral neuropathy defined alternatively as the presence of at least one neurological abnormality (disturbance of the light touch sensation, abolition of ankle reflex or abolition of knee reflex) or a history of foot ulceration. We evaluated the associations between lower-limb complications and the risk of cancer death in people who have smoked cigarettes regularly (most days for at least a year) and those who have never smoked. Also, we tested the association between lower-limb complications and the risk of cardiovascular and all-cause death. Finally, we used the Fine and Gray method to estimate the subdistribution Hazard ratio for cancer death, while accounting for the competing risk of death from non-cancer causes further to adjusting as in model 2 [29].

Statistics were performed using SAS software, version 9.3 (SAS Institute, Cary, NC, USA, www.sas.com) and Stata software version 15.1 (StataCorp, TX, USA, http://www.stata.com). Two-sided *P* values < 0.05 were considered statistically significant.

## Results

### Clinical characteristics at baseline

Among 11,140 ADVANCE participants, 4733 (42%) were women, 4136 (37%) from Asia, 4862 (44%) from established market economies and 2142 (19%) from eastern Europe. Their mean (SD) age and duration of diabetes were 66 (6) and 7.9 (6.3) years, respectively, and the mean (SD) HbA1c was 7.5 (1.6%) at baseline (Table 1). A history of lower-limb complication was reported at baseline in 4293 (38%) participants: 2439 (22%) with PAD and 2973 (27%) with peripheral neuropathy. PAD and peripheral neuropathy were present simultaneously in 1119 (10%) individuals. Characteristics of participants according to a history of PAD or peripheral neuropathy at baseline are displayed in Table 1.

**Table 1:** Characteristics of participants according to history of peripheral artery disease or peripheral neuropathy at baseline

	Overall	Peripheral artery disease		Peripheral neuropathy	
		No	Yes	No	Yes
Number	11,140	8701	2439 (22)	8167	2973 (27)
Gender, n (%)					
Women	4733 (42)	3816	917 (19)	3580	1153 (24)
Men	6407 (58)	4885	1522 (24)	4587	1820 (28)
Region of origin, n (%)					
Asia	4136 (37)	3755	381 (9)	3426	710 (17)
Established market economies	4862 (44)	3225	1637 (34)	3260	1602 (33)
Eastern Europe	2142 (19)	1721	421 (20)	1481	661 (31)
Age, years: mean (SD)	66 (6)	65 (6)	67 (7)	65 [6]	67 (7)
Duration of diabetes, years: mean (SD)	7.9 (6.3)	7.8 (6.2)	8.4 (6.8)	7.6 (6.2)	8.7 (6.6)
Body mass index, kg/m^2^: mean (SD)	28 (5)	28 (5)	29 (5)	28 (5)	29 (6)
Waist circumference, cm: mean (SD)	98 (13)	98 (13)	102 (13)	97 (13)	102 (13)
Systolic blood pressure, mmHg: mean (SD)	145 (21)	145 (21)	146 (21)	145 (21)	146 (21)
Diastolic blood pressure, mmHg: mean (SD)	81 (11)	81 (11)	80 (10)	81 (11)	80 (11)
Mini-mental state examination					
Overall MMSE score, mean (SD)	28 (2)	29 (2)	28 (2)	29 (2)	28 (2)
Participants with normal MMSE score, n (%)	8689 (78)	6900	1789 (21)	6480	2209 (25)
Participants with reduced MMSE score, n (%)	2451 (22)	1801	650 (27)	1687	764 (31)
Education accomplishment					
Age at completion, years: mean (SD)	18 (7)	19 (7)	18 (7)	19 (7)	18 (7)
Participants with basic education, n (%)	7116 (64)	5809	1307 (18)	5402	1714 (24)
Participants with low education, n (%)	4024 (36)	2892	1132 (28)	2765	1259 (31)
HbA1c, mean (SD)					
(%)	7.5 (1.6)	7.5 (1.6)	7.4 (1.4)	7.5 (1.6)	7.5 (1.5)
mmol/mol	59 (17)	59 (17)	58 (15)	59 (17)	59 (16)
Urinary ACR, µg/mg: median (25^th^, 75^th^ percentiles)	1 (1,2)	1.0 (1.0, 2.0)	1.0 (1.0, 2.0)	1.0 (1.0, 2.0)	1.0 (1.0, 2.0)
eGFR, ml/min/1.73m^2^: mean (SD)	74 (17)	75 (17)	71 (18)	75 (18)	72 (17)
Serum total cholesterol, mmol/l: mean (SD)	5.2 (1.2)	5.2 (1.2)	5.0 (1.1)	5.2 (1.2)	5.1 (1.2)
Serum HDL cholesterol, mmol/l: mean (SD)	1.2 (0.3)	1.3 (0.3)	1.2 (0.3)	1.3 (0.4)	1.2 (0.3)
Serum LDL cholesterol, mmol/l: mean (SD)	3.1 (1.0)	3.1 (1.0)	3.0 (1.0)	3.1 (1.0)	3.0 (1.0)
Serum triglycerides, mmol/l: median (25th, 75th percentiles)	1.6 (1.2, 2.3)	1.6 (1.2, 2.3)	1.6 (1.2, 2.3)	1.6 (1.2, 2.3)	1.6 (1.2, 2.3)
History of complications, n (%)					
Coronary arterial disease	2380 (21)	1694	686 (29)	1611	769 (32)
Cerebrovascular disease	1439 (13)	1107	332 (23)	1099	340 (24)
Diabetic kidney disease	2444 (22)	1771	673 [28]	1699	745 (30)
Diabetic retinopathy	1079 (10)	812	267 (25)	748	331 (31)
Dementia	109 (1)	75	34 (31)	67	42 (39)
History of tobacco smoking, n (%)					
Ever	4674 (42)	3303	1371 (29)	3216	1458 (31)
Current	1682 (15)	1287	395 (23)	1212	470 (28)
History of alcohol drinking, n (%)					
Past	4237 (38)	3015	1222 (29)	2997	1240 (29)
Current	3396 (30)	2433	963 (28)	2368	1028 (30)
History of medication use, n (%)					
Metformin	6752 (61)	5238	1514 (22)	4836	1916 (28)
Insulin therapy	159 (1)	133	26 (16)	118	41 (26)
Antihypertensive treatment	7655 (69)	5908	1747 (23)	5552	2103 (27)
Antiplatelet drugs	5199 (47)	3914	1285 [25]	3694	1505 (29)
Statins	3146 (28)	2207	939 (30)	2121	1025 [33]
Study allocations, n (%)					
Intensive glucose control group	5571 (50)	4334	1237 (22)	4063	1508 (27)
Perindopril/indapamide combination	5569 (50)	4365	1204 (22)	4070	1499 (27)

Categorical parameters are expressed as numbers (and percentages for participants with baseline history of PAD or peripheral neuropathy). *ACR* albumin to creatinine ratio. *eGFR* estimated Glomerular Filtration Rate computed by the Chronic Kidney Disease Epidemiology Collaboration equation. Mini-Mental State Examination (MMSE) score: normal (≥ 28), reduced (< 28). Education accomplishment (age at completion of the highest level of formal education): basic (≥ 16 years), low (≤ 15 years)

### Incidence of cancer death

Cancer death occurred in 316 (2.8%) participants during a median of 5.0 (25^th^–75^th^ percentile, 4.7–5.1) years of follow-up corresponding to 53,550 person-years and an incidence rate of 5.9 (95% CI 5.3–6.6) per 1000 person-years. The cumulative incidence of cancer death was 2.1% in women and 3.4% in men. The baseline mean (SD) age, duration of diabetes, HbA1c, systolic and diastolic blood pressure of individuals who subsequently died from cancer causes were 69 (6) years, 7.9 (6.6) years, 7.6 (6.6%), 146 (22) mmHg and 80 (11) mmHg, respectively (Table 2). Hazard ratio for cancer death according to each individual parameter is shown in Table 3, after adjusting for study allocations.

**Table 2:** Characteristics of participants at baseline according to the incidence of cancer death during follow-up

	Cancer death	
	No	Yes
N	10,824	316 (2.8)
Gender, n (%)		
Women	4633	100 (2.1)
Men	6191	216 (3.4)
Region of origin, n (%)		
Asia	4059	77 (1.9)
Established market economies	4688	174 (3.6)
Eastern Europe	2077	65 (3.0)
Age, years: mean (SD)	66 (6)	69 (6)
Duration of diabetes, years: mean (SD)	7.9 (6.3)	7.9 (6.6)
Body mass index, kg/m^2^: mean (SD)	28 (5)	29 (5)
Waist circumference, cm: mean (SD)	98 (13)	101 (13)
Systolic blood pressure, mmHg: mean (SD)	145 (21)	146 (22)
Diastolic blood pressure, mmHg: mean (SD)	81 [11]	80 [11]
Mini-mental state examination		
Overall MMSE score, mean (SD)	28 (2)	28 (2)
Participants with normal MMSE score, n (%)	8456	233 (2.7)
Participants with reduced MMSE score, n (%)	2368	83 (3.4)
Education accomplishment		
Age at completion, years: mean (SD)	18 (7)	17 (6)
Participants with basic education, n (%)	6941	175 (2.5)
Participants with low education, n (%)	3883	141 (3.5)
HbA1c, mean (SD)		
%	7.5 (1.5)	7.6 (1.6)
mmol/mol	59 (17)	59 (17)
Urinary ACR, µg/mg: median (25th, 75th percentiles)	1 (1,2)	1 (1,2)
eGFR, ml/min/1.73m^2^: mean (SD)	74 (17)	71 (17)
Serum total cholesterol, mmol/l: mean (SD)	5.2 (1.2)	5.0 (1.1)
Serum HDL cholesterol, mmol/l: mean (SD)	1.2 (0.3)	1.2 (0.3)
Serum LDL cholesterol, mmol/l: mean (SD)	3.1 (1.0)	2.9 (1.0)
Serum triglycerides, mmol/l: median (25th, 75th percentiles)	1.6 (1.2, 2.3)	1.6 (1.1, 2.3)
History of complications, n (%)		
Coronary arterial disease	2318	62 (2.6)
Cerebrovascular disease	1401	38 (2.6)
Diabetic kidney disease	2358	86 (3.5)
Diabetic retinopathy	1041	38 (3.5)
Dementia	107	2 (1.8)
History of tobacco smoking, n (%)		
Ever	4501	173 (3.7)
Current	1626	56 (3.3)
History of alcohol drinking, n (%)		
Past	4097	140 (3.3)
Current	3281	115 (3.4)
History of medication use, n (%)		
Metformin	6582	170 (2.5)
Insulin therapy	154	5 (3.1)
Antihypertensive treatment	7448	207 (2.7)
Antiplatelet drugs	5058	141 (2.7)
Statins	3058	88 (2.8)
Study allocations, n (%)		
Intensive glucose control group	5423	148 (2.7)
Perindopril/indapamide combination	5422	147 (2.6)

Categorical parameters are expressed as numbers (and percentages for participants who died from cancer causes)

*ACR* albumin to creatinine ratio. *eGFR* estimated Glomerular Filtration Rate computed by the Chronic Kidney Disease Epidemiology Collaboration equation. Mini-Mental State Examination (MMSE) score: normal (≥ 28), reduced (< 28). Education accomplishment (age at completion of the highest level of formal education): basic (≥ 16 years), low (≤ 15 years)

**Table 3:** Risk of cancer death according to each individual parameter at baseline

	Hazard Ratio	95% CI	*p*
Female sex	0.62	0.49–0.79	< 0.0001
Region of origin: established market economies (vs. Asia)	1.87	1.43–2.44	< 0.0001
Region of origin: Eastern Europe (vs. Asia)	1.68	1.21–2.33	0.002
Region of origin: established market economies (vs. Eastern Europe)	1.11	0.84–1.48	0.46
Age (per 1 SD increase)	1.82	1.63–2.03	< 0.0001
Duration of diabetes (per 1 SD increase)	1.01	0.91–1.13	0.83
Body mass index (per 1 SD increase)	1.08	0.97–1.20	0.14
Waist circumference (per 1 SD increase)	1.23	1.11–1.37	< 0.0001
Systolic blood pressure (per 1 SD increase)	1.07	0.96–1.19	0.23
Diastolic blood pressure (per 1 SD increase)	0.91	0.81–1.02	0.10
Mini-Mental State Examination score (< 28 vs. ≥ 28)	1.30	1.01–1.67	0.04
Education accomplishment (≤ 15 vs. ≥ 16 years)	1.44	1.15–1.80	0.001
HbA1c (per 1 SD increase)	1.04	0.93–1.16	0.45
Urinary albumin to creatinine ratio (per 1SD increase)	1.08	0.97–1.21	0.16
Estimated glomerular filtration rate (per 1 SD increase)	0.82	0.73–0.91	0.0003
Serum total cholesterol (per 1 SD increase)	0.85	0.75–0.95	0.007
Serum HDL cholesterol (per 1 SD increase)	0.88	0.78–0.99	0.03
Serum LDL cholesterol (per 1 SD increase)	0.84	0.75–0.95	0.004
Serum triglycerides (per 1 SD increase)	0.97	0.86–1.09	0.60
History of coronary arterial disease	0.91	0.69–1.20	0.52
History of cerebrovascular disease	0.96	0.68–1.34	0.79
History of diabetic kidney disease	1.38	1.08–1.77	0.01
History of diabetic retinopathy	1.34	0.96–1.89	0.09
History of dementia	0.63	0.16–2.52	0.51
History of current smoking	1.21	0.90–1.61	0.20
History of ever smoking	1.67	1.33–2.08	< 0.0001
History of past alcohol drinking	1.28	1.02–1.60	0.03
History of current alcohol drinking	1.27	1.01–1.60	0.04
Use of Metformin	0.75	0.60–0.93	0.01
Use of insulin therapy	1.17	0.48–2.83	0.72
Use of antihypertensive treatment	0.88	0.70–1.11	0.27
Use of antiplatelet drugs	0.92	0.74–1.15	0.48
Use of statins	0.95	0.75–1.22	0.71

Cox proportional hazards survival regressive analysis for each variable adjusted for study allocations. *SD* standard deviation

### Effects of study treatments on the risk for cancer death

Cancer death occurred in 147 (2.6%) participants assigned to perindopril/indapamide combination versus 169 (3.0%) individuals assigned to placebo (incidence rate: 5.5 [95% CI, 4.6—6.4] *versus* 6.3 [5.4—7.4] per 1000 person-years) with no significant difference between blood pressure treatment groups: HR 0.86 (95% CI, 0.69–1.08). Cancer death occurred in 65 (2.3%) participants [incidence rate 4.8 (3.8–6.2) per 1000 person-years] randomized to both intensive glucose control and active blood pressure treatment *versus* 86 (3.1%) individuals [incidence rate 6.4 (5.2–8.0) per 1000 person-years] assigned to standard glucose control and placebo group [HR 0.75 (0.54–1.03)].

### Risk of cancer death according to history of lower-limb complications at baseline

**Fig. 1:**
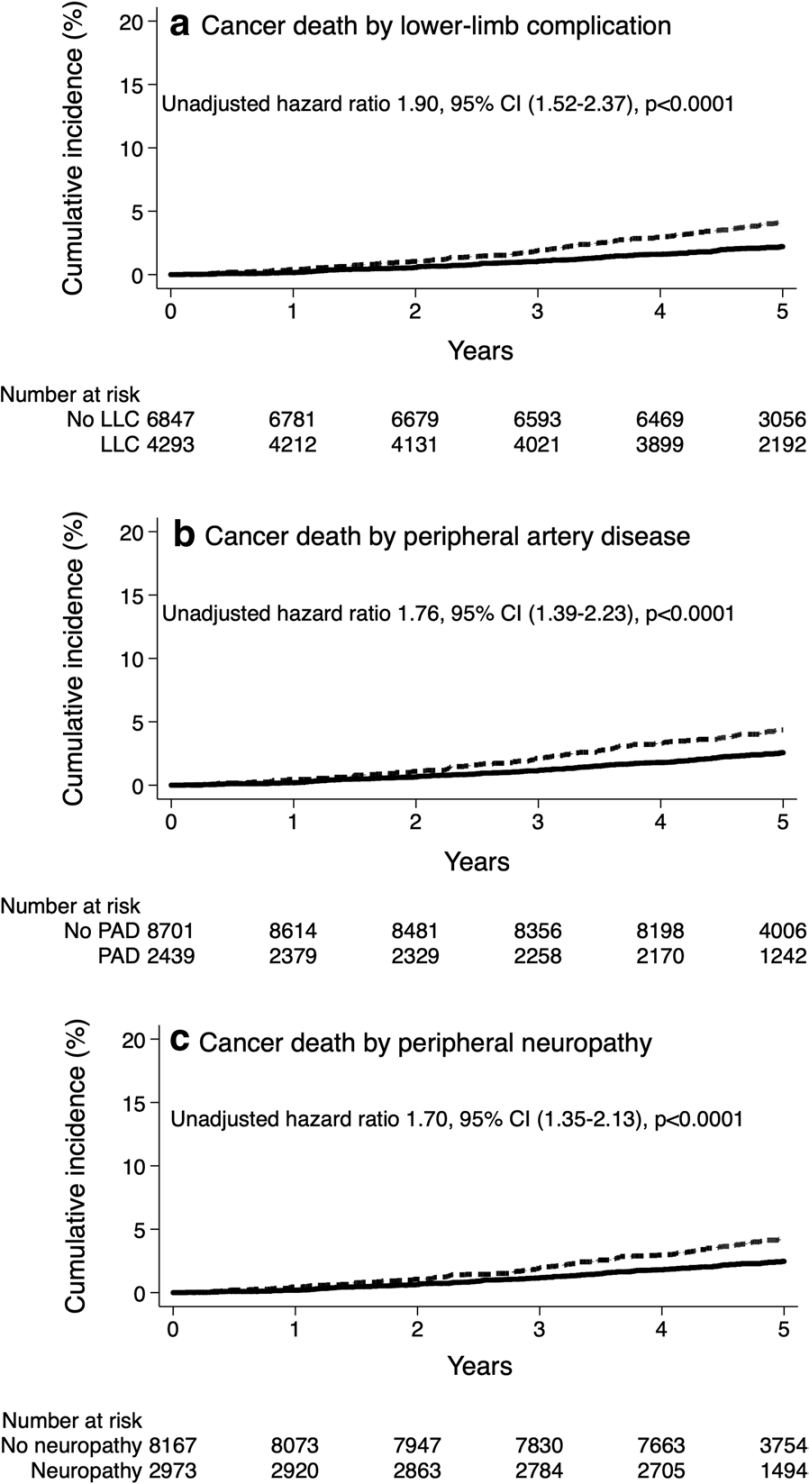
Cumulative incidence of cancer death during follow-up according to history of lower-limb complications at baseline. Solid line: absence of complication; dashed line: presence of complication. *P *values (log-rank test) for comparing absence and presence < 0.0001 for all three complications. *LLC *lower-limb complications, *PAD *peripheral artery disease

The cumulative incidences (Table 4 and Fig. 1) and incidence rates of cancer death were higher in participants with a baseline history of lower-limb complications [8.3 (95% CI, 7.2–9.7] vs. 4.4 (3.7–5.2) per 1000 person-years], PAD [8.9 (7.3–10.8) vs. 5.1 (4.4–5.8) per 1000 person-years] or peripheral neuropathy [8.4 (7.1–10.1) vs. 5.0 [4.3–5.7] per 1000 person-years), compared with individuals without this complication. The risk of cancer death was higher in patients with a baseline history of lower-limb complication [HR 1.62 (1.29–2.04), *p* < 0.0001], PAD [1.43 (1.12–1.83), *p* = 0.004] or peripheral neuropathy [1.49 (1.19–1.88), *p* = 0.0006], compared with those without these conditions after adjusting for age (and its square), sex, region of origin and study allocations. These associations remained significant after further adjustment for a wide range of potential confounders (model 2, Table 4). When we used our alternative definition of lower-limb complication, PAD and peripheral neuropathy remained associated with increased risk of cancer death, but not the history of tissue or limb loss (Additional file 1: Table S2). Comparable results were also observed when peripheral neuropathy was defined as the presence of at least one neurological abnormality or a history of foot ulceration (Additional file 1: Table S3). Associations between lower-limb complications and risk of cancer death were comparable when were stratified analyses by smoking status, with no evidence for statistical interaction (Additional file 1: Table S4). No statistical interaction was observed between PAD and peripheral neuropathy in their association with cancer death (*p* = 0.14).

**Table 4:** Risk of cancer death according to history of lower-limb complications at baseline

		Cancer death, n (%)		Model 1		Model 2	
		No, n	Yes, n (%)	Hazard ratio (95% CI)	*p*	Hazard ratio (95% CI)	*p*
Lower-limb complication	No	6702	145 (2.1)	1.62 (1.29–2.04)	< 0.0001	1.53 (1.21–1.94)	0.0004
	Yes	4122	171 (4.0)				
Peripheral arterial disease	No	8488	213 (2.4)	1.43 (1.12–1.83)	0.004	1.32 (1.02–1.70)	0.03
	Yes	2336	103 (4.2)				
Peripheral neuropathy	No	7971	196 (2.4)	1.49 (1.19–1.88)	0.0006	1.41 (1.11–1.79)	0.004
	Yes	2853	120 (4.0)				

Model 1: age (and its square), sex, region of origin, and study allocations

Model 2: model 1 plus duration of diabetes, body mass index (and its square), waist circumference (and its square), systolic and diastolic blood pressure (and their squares), HbA1c (and its square), urinary ACR, eGFR (and its square), total cholesterol, HDL cholesterol, triglycerides, MMSE score, education accomplishment, history of ever or current smoking, history of past or current alcohol drinking, history of CAD, cerebrovascular disease, diabetic retinopathy or dementia, and use of metformin, insulin, antihypertensive, statin or antiplatelet therapy

### Risk of cancer death according to baseline history of lower-limb complications considering death from non-cancer causes as a competing risk

All-cause and cardiovascular death occurred in 1031 (9.2%) and 542 (4.9%) participants during follow-up. Their incidence rates were 19.2 (95% CI 18.1–20.5) and 10.1 (9.3–11.0) per 1000 person-years, respectively. We observed an increased risk of all-cause and cardiovascular death in patients with a baseline history of lower-limb complications or PAD, compared with those without these complications (Additional file 1: Table S5). The history of peripheral neuropathy was associated with increased risk of all-cause, but not cardiovascular, death (Additional file 1: Table S5). The association between lower-limb complications and cancer death did not change substantially when we considered death from non-cancer causes as a competing risk (Additional file 1: Table S5).

### Risk of incident cancers according to history of lower-limb complications at baseline

Incident cancers were diagnosed in 700 (6.3%) participants during follow-up (Additional file 1: Table S1), corresponding to 52,473 person-years and an incidence rate of 13.3 (95% CI, 12.4–14.4) per 1000 person-years. The cumulative incidences (Table 5) and incidence rates of cancers were higher in participants with a baseline history of lower-limb complication [17.1 (95% CI, 15.4–19.0) vs.11.0 (9.9–12.2) per 1000 person-years], PAD [18.9 (16.5–21.6) vs. 11.8 (10.8–12.9) per 1000 person-years] or peripheral neuropathy [16.7 (14.7–19.0) vs. 12.1 (11.1–13.3) per 1000 person-years], compared with those without these conditions. The relative risk of incident cancers was higher in participants with (vs. without) a baseline history of lower-limb complications [HR 1.23 (1.05–1.44), *p* = 0.01] or PAD (1.20 (1.01–1.43), *p* = 0.04], but not those with peripheral neuropathy [1.14 (0.97–1.35), *p* = 0.11] after adjustment for cofounding variables as in model 2 (Table 5). Association with PAD was particularly observed for solid and digestive malignant neoplasms (Additional file 1: Table S6). Based on the alternative definitions of lower-limb complications, PAD remained associated with excess risk of incident cancers, while peripheral neuropathy and tissue or limb loss did not (Additional file 1: Table S2). The history of diabetic neuropathy, defined as the presence of at least one neurological abnormality or a history of foot ulceration, was not associated with the risk of incident cancers (Additional file 1: Table S3).

**Table 5:** Risk of incident cancers according to history of lower-limb complications at baseline

		Incident cancers, n (%)		Model 1		Model 2	
		No, n	Yes, n (%)	Hazard ratio (95% CI)	*p*	Hazard ratio (95% CI)	*p*
Lower-limb complication	No	6490	357 (5.2)	1.34 (1.15–1.56)	0.0001	1.23 (1.05–1.44)	0.01
	Yes	3950	343 (8.0)				
Peripheral arterial disease	No	8214	487 (5.6)	1.32 (1.12–1.56)	0.001	1.20 (1.01–1.43)	0.04
	Yes	2226	213 (8.7)				
Peripheral neuropathy	No	7699	468 (5.7)	1.24 (1.06–1.45)	0.008	1.14 (0.97–1.35)	0.11
	Yes	2741	232 (7.8)				

Model 1: age (and its square), sex, region of origin, and study allocations

Model 2: model 1 plus duration of diabetes, body mass index (and its square), waist circumference (and its square), systolic and diastolic blood pressure (and their squares), HbA1c (and its square), urinary ACR, eGFR (and its square), total cholesterol, HDL cholesterol, triglycerides, MMSE score, education accomplishment, history of ever or current smoking, history of past or current alcohol drinking, history of CAD, cerebrovascular disease, diabetic retinopathy or dementia, and use of metformin, insulin, antihypertensive, statin or antiplatelet therapy

## Discussion

In the present study, we investigated the relationship between a baseline history of lower-limb complications and the risk of cancer death in patients with type 2 diabetes in the ADVANCE study. We observed an independent association between the history of lower-limb complications, both PAD and peripheral neuropathy, and excess 5-year risk of cancer death. No statistical interaction was observed between PAD and sensory peripheral neuropathy in their association with the risk of cancer death. Our findings were reliable when we considered death from non-cancer causes as a competing risk. Of note, lower-limb complications and PAD were associated with increased risk of both all-cause and cardiovascular death, while neuropathy was associated with only all-cause death. The history of PAD, but not peripheral neuropathy, was also associated with a higher incidence of cancers, mainly solid and digestive neoplasms.

### Lower-limb complications and risk of cancer death

As far as we know, this is the first report of increased risk of cancer death related to lower-limb complications in patients with type 2 diabetes. Few previous studies reported increased incidence of cancers among patients with intermittent claudication or critical limb ischemia in general population [30, 31]. A recent study has shown an association between lower-limb arterial thrombosis and increased risk of subsequent cancers in 6600 patients (12% with diabetes) from Danish nationwide population-based registries [32]. A lower-limb arterial thrombosis was also associated with a high risk of all-cause mortality following some site-specific (mainly smoking-dependent) cancers.

### Potential mechanisms linking lower-limb complications to cancer death

Our findings cannot allow any etiological conclusion, and may only suggest lower-limb complications as proxy for cancer death. Indeed, lower-limb complications are surrogates for advanced microvascular and macrovascular disease, and may lead to a greater likelihood of multisystem disease. Nevertheless, lower-limb complications and malignancies may share some modifiable risk factors, including age, obesity and tobacco smoking [33–35]. These risk factors are unlikely to explain our findings as the key observed associations were reliable after adjustment for these confounders. Furthermore, the magnitude of the associations between lower-limb complications and cancer death were comparable when we performed analyses in people who have smoked cigarettes regularly and those who have never smoked, without evidence for statistical interaction. Further studies are needed to investigate the potential contribution of smoking in the relationship between lower-limb complications and cancer death. Lower-limb complications and malignancies may also share some common biological disorders including chronic inflammation, increased advanced glycation endproducts and oxidative stress [36–39]. Oxidative stress leads to lipid peroxidation and DNA damage, which are involved in microvascular disease, atherosclerosis and cancer [36, 40]. Furthermore, endothelial dysfunction, an important impairment involved in lower-limb complications, has also been linked to increased risk of solid-tumor cancer [41]. While lower-limb complications, involving microvascular disease and atherosclerosis (as a result at least partly of early endothelial dysfunction), cancer is mainly characterized by excessive angiogenesis, which may be driven by endothelial metabolic aberrations [42].

### Effects of glucose and blood pressure controls in risk of cancer death

We did not observe an association between ADVANCE allocation to blood pressure treatment and the risk of cancer death, as was previously reported for intensive glucose control [28]. However, we have observed a non-significant decrease in risk of cancer death in participants assigned to both glucose control and blood pressure interventions, compared with those allocated to conventional glucose control and placebo blood pressure treatments. This observation may raise the hypothesis wondering the potential effect of multifactorial intervention on the incidence of cancer death in people with type 2 diabetes. Randomised clinical trials are required to address this hypothesis. Of note, Rasmussen-Torvik and coworkers reported an inverse association between the number of ideal cardiovascular health metrics, as defined by the American Heart Association [43] (including blood pressure and glycemia) and combined cancer incidence in the Atherosclerosis Risk In Communities (ARIC) cohort [44].

### Strengths and limitations

The main strength of our work is the use of a large international multicentre study of 11,140 patients with type 2 diabetes, with a comprehensive range of data including demographic, clinical and biological features at baseline. Participants were also prospectively followed for 5 years, with adjudicated causes of mortality, including cancer death within a central and independent adjudication committee process. However, incident cancers were collected from case report forms and reports of serious adverse events, without adjudication or validation using cancer registry data. The other limitation of our study is related to the post hoc analyses of a randomized controlled trial, and the use of a clinical trial population, which may not be representative of all patients with type 2 diabetes. Also, we used a pragmatic assessment of lower-limb complications without a prespecified research protocol, and we missed some data regarding PAD (intermittent claudication, ankle brachial index, toe pressure or transcutaneous oxygen pressure) or peripheral sensory neuropathy (symptoms or vibration sensation test). We do not believe that these issues influenced our findings as we investigated a widespread spectrum of data including clinical examination (pulses palpation, light touch sensation and reflex tests), condition (foot ulceration) and procedures (revascularisation and lower-extremity amputation). However, we cannot exclude that non-diabetic causes of neuropathy could have biased our findings, especially neurotoxicity of cancer treatment (chemotherapeutic drugs and radiotherapy), paraneoplastic neurologic disorders or nervous dysfunction caused by the cancer [45]. Nevertheless, cancer-related neuropathy seemed to be unlikely, since we dealt with a high prevalent neuropathy (n = 2973, 27%) consistent with diabetic origin, while incident cancers were diagnosed only in 700 (6%) participants. Finally, our findings were not controlled for socioeconomic position as we missed appropriate data, except for education accomplishment.

## Conclusions

In summary, our study links PAD and sensory peripheral neuropathy to excess 5-year risk for cancer death in patients with type 2 diabetes. In addition, PAD was also associated with increased risk of incident cancers, especially solid and digestive ones. Our findings provide new evidence on the non-cardiovascular prognostic burden of lower-limb complications in people with type 2 diabetes, and encourage continuing careful evaluation and management of cancers in this population.

## Supplementary Information

**Additional file 1: Table S1.** Site-specific cancers and their frequencies in the ADVANCE study. **Table S2. **Risk of cancer death and incident cancers according to history of lower-limb complications (using an alternative definition) at baseline. **Table S3. **Risk of cancer death and incident cancers according to history of diabetic neuropathy (using an alternative definition) at baseline. **Table S4. **Risk of cancer death according to history of lower-limb complications by smoking status at baseline. **Table S5. **Risks of cardiovascular, all-cause and cancer (considering death from non-cancer causes as a competing risk) death according to history of lower-limb complications at baseline. **Table S6.** Risk of site-specific cancers according to history of lower-limb complications at baseline

## Data Availability

The datasets analysed during the current study are not publicly available due to consideration of intellectual property, due to many ongoing active collaborations worldwide, and to continuing analyses by the study investigators, but may be available from the principal investigator on reasonable request.

## Abbreviations

ADVANCE: Action in Diabetes and Vascular Disease: PreterAx and DiamicroN Modified-Release Controlled Evaluation
ACR: Albumin-to-creatinine ratio
ARIC: Atherosclerosis Risk In Communities cohort
CI: Confidence interval
DKD: Diabetic Kidney Disease
eGFR: Estimated Glomerular Filtration Rate
HR: Hazard ratio
ICD-10: International Classification of Diseases Code Tenth Revision
MMSE: Mini-Mental State Examination
PAD: Peripheral Artery Disease
SD: Standard Deviation
