# Supplementary material for: History of lower-limb complications and risk of cancer death in people with type 2 diabetes
Source: Cardiovasc Diabetol. 2021 Jan 4;20:3. doi: 10.1186/s12933-020-01198-y (PMC7784290; doi:10.1186/s12933-020-01198-y)
Supplement: Supplementary file 1 — Additional file 1: Table S1. Site-specific cancers and their frequencies in the ADVANCE study. Table S2. Risk of cancer death and incident cancers according to history of lower-limb complications (using an alternative definition) at baseline. Table S3. Risk of cancer death and incident cancers according to history of diabetic neuropathy (using an alternative definition) at baseline. Table S4. Risk of cancer death according to history of lower-limb complications by smoking status at baseline. Table S5. Risks of cardiovascular, all-cause and cancer (considering death from non-cancer causes as a competing risk) death according to history of lower-limb complications at baseline. Table S6. Risk of site-specific cancers according to history of lower-limb complications at baseline [file 12933_2020_1198_MOESM1_ESM.docx]

**History of lower-limb complications and risk of cancer death**

**in people with type 2 diabetes**

Kamel Mohammedi, Stephen Harrap, Giuseppe Mancia, Michel Marre, Neil Poulter**,**

John Chalmers, Mark Woodward

**Additional Material**

**Additional Table 1.** Site-specific cancers and their frequencies in the ADVANCE study

**Additional Table 2.** Risk of cancer death and incident cancers according to history of lower-limb complications (using an alternative definition) at baseline

**Additional Table 3.** Risk of cancer death and incident cancers according to history of diabetic neuropathy (using an alternative definition) at baseline

**Additional Table 4.** Risk of cancer death according to history of lower-limb complications by smoking status at baseline

**Additional Table 5.** Risks of cardiovascular, all-cause and cancer (considering death from non-cancer causes as a competing risk) death according to history of lower-limb complications at baseline

**Additional Table 6.** Risk of site-specific cancers according to history of lower-limb complications at baseline

**Additional Table 1. Site-specific cancers and their frequencies in the ADVANCE study**

| **ICD-10 codes** | **Cancer sites** | **N (%)** |
| --- | --- | --- |
| **C00-C97** | **All malignant neoplasms** | 700 (6.3) |
| C00-C75 | **Solid malignant neoplasms*** | 631 (5.7) |
| C00-C14 | **Lip, oral cavity and pharynx** | 17 (0.1) |
| C00 | Malignant neoplasm of lip |  |
| C01 | Malignant neoplasm of base of tongue |  |
| C02 | Malignant neoplasm of other and unspecified parts of tongue |  |
| C03 | Malignant neoplasm of gum |  |
| C04 | Malignant neoplasm of floor of mouth |  |
| C05 | Malignant neoplasm of palate |  |
| C06 | Malignant neoplasm of other and unspecified parts of mouth |  |
| C07 | Malignant neoplasm of parotid gland |  |
| C08 | Malignant neoplasm of other and unspecified major salivary glands |  |
| C09 | Malignant neoplasm of tonsil |  |
| C10 | Malignant neoplasm of oropharynx |  |
| C11 | Malignant neoplasm of nasopharynx |  |
| C12 | Malignant neoplasm of pyriform sinus |  |
| C13 | Malignant neoplasm of hypopharynx |  |
| C14 | Malignant neoplasm of other and ill-defined sites in the lip- oral cavity and pharynx |  |
| C15-C26 | **Digestive organs** | 222 (2) |
| C15 | Malignant neoplasm of oesophagus |  |
| C16 | Malignant neoplasm of stomach |  |
| C17 | Malignant neoplasm of small intestine |  |
| C18 | Malignant neoplasm of colon |  |
| C19 | Malignant neoplasm of rectosigmoid junction |  |
| C20 | Malignant neoplasm of rectum |  |
| C21 | Malignant neoplasm of anus and anal canal |  |
| C22 | Malignant neoplasm of liver and intrahepatic bile ducts |  |
| C23 | Malignant neoplasm of gallbladder |  |
| C24 | Malignant neoplasm of other and unspecified parts of biliary tract |  |
| C25 | **Malignant neoplasm of pancreas** | 32 (0.3) |
| C26 | Malignant neoplasm of other and ill-defined digestive organs |  |
| C30-C39 | **Respiratory and intrathoracic organs** | 116 (1) |
| C30 | Malignant neoplasm of nasal cavity and middle ear |  |
| C31 | Malignant neoplasm of accessory sinuses |  |
| C32 | Malignant neoplasm of larynx |  |
| C33 | Malignant neoplasm of trachea |  |
| C34 | Malignant neoplasm of bronchus and lung |  |
| C37 | Malignant neoplasm of thymus |  |
| C38 | Malignant neoplasm of heart- mediastinum and pleura |  |
| C39 | Malignant neoplasm of other and ill-defined sites in the respiratory system and intrathoracic organs |  |
| C50 | **Malignant neoplasm of breast** | 64 (0.6) |
| C51-C58 | **Female genital organs** | 16 (0.1) |
| C51 | Malignant neoplasm of vulva |  |
| C52 | Malignant neoplasm of vagina |  |
| C53 | Malignant neoplasm of cervix uteri |  |
| C54 | Malignant neoplasm of corpus uteri |  |
| C55 | Malignant neoplasm of uterus- part unspecified |  |
| C56 | Malignant neoplasm of ovary |  |
| C57 | Malignant neoplasm of other and unspecified female genital organs |  |
| C58 | Malignant neoplasm of placenta |  |
| C60-C63 | **Male genital organs** | 86 (0.8) |
| C60 | Malignant neoplasm of penis |  |
| C61 | Malignant neoplasm of prostate |  |
| C62 | Malignant neoplasm of testis |  |
| C63 | Malignant neoplasm of other and unspecified male genital organs |  |
| C81-C96 | **Malignant neoplasms, stated or presumed to be primary, of lymphoid, haematopoietic and related tissue** | 40 (0.4) |
| C81 | Hodgkin's disease |  |
| C82 | Follicular [nodular] non-Hodgkin's lymphoma |  |
| C83 | Diffuse non-Hodgkin's lymphoma |  |
| C84 | Peripheral and cutaneous T-cell lymphomas |  |
| C85 | Other and unspecified types of non-Hodgkin's lymphoma |  |
| C88 | Malignant immunoproliferative diseases |  |
| C90 | Multiple myeloma and malignant plasma cell neoplasms |  |
| C91 | Lymphoid leukaemia |  |
| C92 | Myeloid leukaemia |  |
| C93 | Monocytic leukaemia |  |
| C94 | Other leukaemias of specified cell type |  |
| C95 | Leukaemia of unspecified cell type |  |
| C96 | Other and unspecified malignant neoplasms of lymphoid- haematopoietic and related tissue |  |
| Cancers were diagnosed according to the International Classification of Diseases Code, Tenth Revision (ICD-10). *Solid malignant neoplasms stated or presumed to be primary, of specified sites, except of lymphoid, haematopoietic and related tissue. | | |

**Additional Table 2. Risk of cancer death and incident cancers according to history of lower-limb complications (using an alternative definition†) at baseline**

|  |  | **Cancer death** | | **Hazard ratio**  **(95% CI)** | **Incident cancers** | | **Hazard ratio**  **(95% CI)** |
| --- | --- | --- | --- | --- | --- | --- | --- |
|  |  | **No, n** | **Yes, n (%)** |  | **No, n** | **Yes, n (%)** |  |
| **Peripheral arterial disease** | No | 8589 | 216 (2.4) | 1.33 (1.03 – 1.72) | 8313 | 492 (5.6) | 1.22 (1.03 – 1.45) |
|  | Yes | 2235 | 100 (4.3) |  | 2127 | 208 (8.9) |  |
| **Peripheral neuropathy** | No | 8071 | 197 (2.4) | 1.46 (1.15 – 1.85) | 7792 | 476 (5.8) | 1.13 (0.96 – 1.33) |
|  | Yes | 2573 | 119 (4.1) |  | 2648 | 224 (7.8) |  |
| **Tissue or limb loss** | No | 10508 | 306 (2.8) | 0.92 (0.48 – 1.73) | 10137 | 677 (6.3) | 1.01 (0.66 – 1.52) |
|  | Yes | 316 | 10 (3.2) |  | 303 | 23 (7.1) |  |
| Adjusted as in model 2: age (and its square), sex, region of origin, study allocations, duration of diabetes, body mass index (and its square), waist circumference (and its square), systolic and diastolic blood pressure (and their squares), HbA1c (and its square), urinary ACR, eGFR (and its square), total cholesterol, HDL cholesterol, triglycerides, MMSE score, education accomplishment, history of ever or current smoking, history of past or current alcohol drinking, history of CAD, cerebrovascular disease, diabetic retinopathy or dementia, and use of metformin, insulin, antihypertensive, statin or antiplatelet therapy.  **†**PAD: lack of peripheral pulse palpation or requirement of lower-limb revascularisation; peripheral neuropathy: at least two neurological abnormalities: disturbance of the light touch sensation, abolition of ankle or knee reflex; tissue or limb loss: foot ulceration or lower-extremity amputation. | | | | | | | |

**Additional Table 3. Risk of cancer death and incident cancers according to history of diabetic neuropathy (using an alternative definition*) at baseline**

|  |  | **Cancer death** | | **Hazard ratio**  **(95% CI)** | **Incident cancers** | | **Hazard ratio**  **(95% CI)** |
| --- | --- | --- | --- | --- | --- | --- | --- |
|  |  | **No, n** | **Yes, n (%)** |  | **No, n** | **Yes, n (%)** |  |
| **Peripheral arterial disease** | No | 7672 | 185 (2.3) | 1.40 (1.11 – 1.77) | 7417 | 440 (5.6) | 1.17 (0.99 – 1.38) |
|  | Yes | 3152 | 131 (4.0) |  | 3023 | 260 (7.9) |  |
| Adjusted as in model 2: age (and its square), sex, region of origin, study allocations, duration of diabetes, body mass index (and its square), waist circumference (and its square), systolic and diastolic blood pressure (and their squares), HbA1c (and its square), urinary ACR, eGFR (and its square), total cholesterol, HDL cholesterol, triglycerides, MMSE score, education accomplishment, history of ever or current smoking, history of past or current alcohol drinking, history of CAD, cerebrovascular disease, diabetic retinopathy or dementia, and use of metformin, insulin, antihypertensive, statin or antiplatelet therapy.  *The presence of at least one neurological abnormality (disturbance of the light touch sensation, abolition of ankle reflex or abolition of knee reflex) or a history of foot ulceration. | | | | | | | |

**Additional Table 4. Risk of cancer death according to history of lower-limb complications by smoking status at baseline**

|  | **Never smokers** | | | | **Smokers^£^** | | |  |
| --- | --- | --- | --- | --- | --- | --- | --- | --- |
|  |  | **Cancer death** | | **Hazard ratio**  **(95% CI)** | **Cancer death** | | **Hazard ratio**  **(95% CI)** | ***P***  ***for interaction*** |
|  |  | **No, n** | **Yes, n (%)** |  | **No, n** | **Yes, n (%)** |  |  |
| **Lower-limb complications** | No | 4297 | 76 (1.7) | 1.57 (1.11 – 2.24) | 2405 | 69 (2.8) | 1.51 (1.09 – 2.09) | 0.91 |
|  | Yes | 2026 | 67 (3.2) |  | 2096 | 104 (4.7) |  |  |
| **Peripheral arterial disease** | No | 5289 | 109 (2.0) | 1.26 (0.84 – 1.91) | 3199 | 104 (3.1) | 1.35 (0.98 – 1.87) | 0.83 |
|  | Yes | 1034 | 34 (3.2) |  | 1302 | 69 (5.0) |  |  |
| **Peripheral neuropathy** | No | 4853 | 98 (2.0) | 1.32 (0.91 – 1.92) | 3118 | 98 (3.1) | 1.50 (1.10 – 2.06) | 0.54 |
|  | Yes | 1470 | 45 (3.0) |  | 1383 | 75 (5.1) |  |  |
| Adjusted for age (and its square), sex, region of origin, study allocations, duration of diabetes, body mass index (and its square), waist circumference (and its square), systolic and diastolic blood pressure (and their squares), HbA1c (and its square), urinary ACR, eGFR (and its square), total cholesterol, HDL cholesterol, triglycerides, MMSE score, education accomplishment, history of past or current alcohol drinking, history of CAD, cerebrovascular disease, diabetic retinopathy or dementia, and use of metformin, insulin, antihypertensive, statin or antiplatelet therapy.  Interaction between smoking and lower-limb complications in their association with cancer death.  ^£^people who smoked cigarettes regularly (on most days for at least a year). | | | | | | | | |

**Additional Table 5. Risks of cardiovascular, all-cause and cancer (considering death from non-cancer causes as a competing risk) death according to history of lower-limb complications at baseline**

|  |  | **Cardiovascular death** | | | **All-cause death** | | | **Cancer death‡** |
| --- | --- | --- | --- | --- | --- | --- | --- | --- |
|  |  | **No, n** | **Yes, n (%)** | **Hazard ratio**  **(95% CI)** | **No, n** | **Yes, n (%)** | **Hazard ratio**  **(95% CI)** | **SHR**  **(95% CI)** |
| **Lower-limb complication** | No | 6585 | 262 (3.8) | 1.27  (1.06 – 1.52) | 6360 | 487 (7.1) | 1.37  (1.21 – 1.56) | 1.51  (1.184 - 1.93) |
|  | Yes | 4013 | 280 (6.5) |  | 3749 | 544 (12.7) |  |  |
| **Peripheral arterial disease** | No | 8357 | 344 (3.9) | 1.51  (1.25 – 1.82) | 8034 | 667 (7.7) | 1.45  (1.26 – 1.66) | 1.29  (0.99 - 1.69) |
|  | Yes | 2241 | 198 (8.1) |  | 2075 | 364 (14.9) |  |  |
| **Peripheral neuropathy** | No | 7813 | 354 (4.3) | 1.12  (0.93 – 1.35) | 7512 | 655 (8.0) | 1.26  (1.10 – 1.44) | 1.40  (1.11 - 1.79) |
|  | Yes | 2785 | 188 (6.3) |  | 2597 | 376 (12.6) |  |  |
| Adjusted as in model 2: age (and its square), sex, region of origin, study allocations, duration of diabetes, body mass index (and its square), waist circumference (and its square), systolic and diastolic blood pressure (and their squares), HbA1c (and its square), urinary ACR, eGFR (and its square), total cholesterol, HDL cholesterol, triglycerides, MMSE score, education accomplishment, history of ever or current smoking, history of past or current alcohol drinking, history of CAD, cerebrovascular disease, diabetic retinopathy or dementia, and use of metformin, insulin, antihypertensive, statin or antiplatelet therapy.  **‡**Subdistribution hazard ratios (SHR), with 95% CI, computed after considering death from non-cancer causes as competing risk further to model 2. | | | | | | | | |

**Additional Table 6. Risk of site-specific cancers according to history of lower-limb complications at baseline**

|  |  | Incidence of cancers | |  |
| --- | --- | --- | --- | --- |
| **Cancer sites** |  | No, n | Yes, n (%) | Hazard ratio  (95% CI) |
| **Solid malignant neoplasms** |  |  |  |  |
| Lower-limb complication | No | 6523 | 324 (4.7) | 1.21 (1.03 – 1.43) |
|  | Yes | 3986 | 307 (7.1) |  |
| Peripheral arterial disease | No | 8266 | 435 (5.0) | 1.25 (1.04 – 1.49) |
|  | Yes | 2243 | 196 (8.1) |  |
| Peripheral neuropathy | No | 7740 | 427 (5.2) | 1.11 (0.93 – 1.32) |
|  | Yes | 2769 | 204 (6.9) |  |
| **Lip, oral cavity and pharynx** |  |  |  |  |
| Lower-limb complication | No | 6839 | 8 (0.1) | 1.41 (0.51 – 3.85) |
|  | Yes | 4284 | 9 (0.2) |  |
| Peripheral arterial disease | No | 8688 | 13 (0.1) | 0.85 (0.26 – 2.74) |
|  | Yes | 2435 | 4 (0.2) |  |
| Peripheral neuropathy | No | 8156 | 11 (0.1) | 1.17 (0.41 – 3.33) |
|  | Yes | 2967 | 6 (0.2) |  |
| **Digestive organs** |  |  |  |  |
| Lower-limb complication | No | 6734 | 113 (1.6) | 1.31 (0.99 – 1.73) |
|  | Yes | 4184 | 109 (2.5) |  |
| Peripheral arterial disease | No | 8552 | 149 (1.7) | 1.49 (1.10 – 2.00) |
|  | Yes | 2366 | 73 (3.0) |  |
| Peripheral neuropathy | No | 8014 | 153 (1.9) | 1.06 (0.79 – 1.42) |
|  | Yes | 2904 | 69 (2.3) |  |
| **Pancreas** |  |  |  |  |
| Lower-limb complication | No | 6832 | 15 (0.2) | 1.31 (0.62 – 2.77) |
|  | Yes | 4276 | 17 (0.4) |  |
| Peripheral arterial disease | No | 8682 | 19 (0.2) | 1.78 (0.82 – 3.87) |
|  | Yes | 2426 | 13 (0.5) |  |
| Peripheral neuropathy | No | 8145 | 22 (0.3) | 0.88 (0.39 – 1.99) |
|  | Yes | 2963 | 10 (0.3) |  |
| **Respiratory and intrathoracic organs** |  |  |  |  |
| Lower-limb complication | No | 6792 | 55 (0.8) | 1.40 (0.95 – 2.07) |
|  | Yes | 4232 | 61 (1.4) |  |
| Peripheral arterial disease | No | 8626 | 75 (0.9) | 1.41 (0.94 – 2.13) |
|  | Yes | 2398 | 41 (1.7) |  |
| Peripheral neuropathy | No | 8094 | 73 (0.9) | 1.37 (0.92 – 2.03) |
|  | Yes | 2930 | 43 (1.4) |  |
| **Breast** |  |  |  |  |
| Lower-limb complication | No | 6811 | 36 (0.5) | 1.39 (0.82 – 2.36) |
|  | Yes | 4265 | 28 (0.6) |  |
| Peripheral arterial disease | No | 8653 | 48 (0.5) | 1.26 (0.69 – 2.33) |
|  | Yes | 2423 | 16 (0.7) |  |
| Peripheral neuropathy | No | 8119 | 48 (0.6) | 0.98 (0.55 – 1.77) |
|  | Yes | 2957 | 16 (0.5) |  |
| **Female genital organs** |  |  |  |  |
| Lower-limb complication | No | 6839 | 8 (0.1) | 1.90 (0.66 – 5.51) |
|  | Yes | 4285 | 8 (0.2) |  |
| Peripheral arterial disease | No | 8691 | 10 (0.1) | 2.93 (0.97 – 8.83) |
|  | Yes | 2433 | 6 (0.2) |  |
| Peripheral neuropathy | No | 8155 | 12 (0.1) | 0.93 (0.29 – 3.03) |
|  | Yes | 2969 | 4 (0.1) |  |
| **Male genital organs** |  |  |  |  |
| Lower-limb complication | No | 6805 | 42 (0.6) | 1.04 (0.66 – 1.64) |
|  | Yes | 4249 | 44 (1.0) |  |
| Peripheral arterial disease | No | 8643 | 58 (0.7) | 1.01 (0.62 – 1.64) |
|  | Yes | 2411 | 28 (1.1) |  |
| Peripheral neuropathy | No | 8115 | 52 (0.6) | 1.26 (0.80 – 1.98) |
|  | Yes | 2939 | 34 (1.1) |  |
| **Lymphoid, haematopoietic and related tissue** |  |  |  |  |
| Lower-limb complication | No | 6827 | 20 (0.3) | 1.30 (0.68 – 2.51) |
|  | Yes | 4273 | 20 (0.5) |  |
| Peripheral arterial disease | No | 8672 | 29 (0.3) | 0.98 (0.47 – 2.05) |
|  | Yes | 2428 | 11 (0.4) |  |
| Peripheral neuropathy | No | 8140 | 27 (0.3) | 1.15 (0.58 – 2.27) |
|  | Yes | 2960 | 13 (0.4) |  |
| Adjusted as in model 2: age (and its square), sex (except for female and male genital organs), region of origin, study allocations, duration of diabetes, body mass index (and its square), waist circumference (and its square), systolic and diastolic blood pressure (and their squares), HbA1c (and its square), urinary ACR, eGFR (and its square), total cholesterol, HDL cholesterol, triglycerides, MMSE score, education accomplishment, history of ever or current smoking, history of past or current alcohol drinking, history of CAD, cerebrovascular disease, diabetic retinopathy or dementia, and use of metformin, insulin, antihypertensive, statin or antiplatelet therapy. | | | | |
